# Umbilical cord-care practices in low- and middle-income countries: a systematic review

**DOI:** 10.1186/s12884-017-1250-7

**Published:** 2017-02-20

**Authors:** Patricia S. Coffey, Siobhan C. Brown

**Affiliations:** PATH, 2201 Westlake Avenue, Suite 200, Seattle, WA 98121 USA

**Keywords:** Neonatology, Umbilical cord care, Low- and middle-income countries, Behavior change

## Abstract

**Background:**

Neonatal sepsis is the third leading cause of deaths for infants in their first month of life. The newly cut umbilical cord can be a pathway for bacteria that can cause newborn sepsis and death. Optimal umbilical cord care practices for newborns and during the first week of life, especially in settings with poor hygiene, has the potential to avoid these preventable neonatal deaths. The purpose of this review of cord care practices is to assist in the development of behavior-change strategies to support introduction of novel cord-care regimens, particularly 7.1% chlorhexidine digluconate for umbilical cord care.

**Methods:**

We searched domestic and international databases for articles that were published in English between January 1, 2000, and August 24, 2016. We found 321 articles and reviewed 65 full-text articles using standardized inclusion criteria. The primary criteria for inclusion was a description of substances applied to the umbilical cord stump in the days following birth.

**Results:**

We included 46 articles in this review of umbilical cord-care practices. Articles included data from 15 low- and middle-income countries in sub-Saharan Africa (8 countries), Asia (5 countries), North Africa (1 country), and Latin America and the Caribbean (1 country). Findings from this review suggest that documentation of cord-care practices is not consistent throughout low- and middle-income countries, yet existing literature depicts a firm tradition of umbilical cord care in every culture. Cord-care practices vary by country and by regions or cultural groups within a country and employ a wide range of substances. The desire to promote healing and hasten cord separation are the underlying beliefs related to application of substances to the umbilical cord. The frequency of application of the substance (either the number of days or the number of times per day the substance was applied), and source and cost of products used is not well-characterized.

**Conclusions:**

This desire to actively care for the umbilical cord of a newborn—as noted in the variety of cord care practices and beliefs identified in this review—points toward the need to contextualize any behavior change approach to align with the local culture.

**Electronic supplementary material:**

The online version of this article (doi:10.1186/s12884-017-1250-7) contains supplementary material, which is available to authorized users.

## Background

Neonatal sepsis is responsible for more than 15% of neonatal deaths worldwide [1] and is the third leading cause of deaths for infants in their first month of life. A newly cut umbilical cord can be a pathway for bacteria that can cause newborn sepsis and death.. Optimal umbilical cord care practices for newborns and during the first week of life, especially in settings with poor hygiene, has the potential to avoid these preventable neonatal deaths.

Harmful traditional cord-care practices are often cited as an important public health concern [2, 3]. A clear understanding of behavioral intention underlying traditional cord care practices in low- and middle-income countries can be helpful in addressing high rates of neonatal sepsis. Although systematic evidence reviews of cord-cleansing practices have been conducted previously [4, 5], the qualitative nature of cord-care practices has not been summarized to-date. This review fills a gap in the literature by systematically reviewing available evidence related to traditional cord-care practices and assessing the likely impact of product categories on infection risk.

## Methods

Our initial search focused on studies that described traditional umbilical cord care practices globally. For the purposes of this article, traditional practices were those that focused on the cultural beliefs and customs that guided how the umbilical cord was cared for, including the length of the cord stump, substances applied, and the decisions regarding disposal of the cord stump. We developed systematic searches for PubMed and Google Scholar using controlled vocabulary (Additional file 1: Search Terminology). Initial criteria for eligibility were determined by topic, time period, and language of the publication. We included articles that were published between January 1, 2000, and January 30, 2016. A second search was performed in August 2016 to account for any publications during the intervening months. The language of publication was limited to English. References in the identified articles were reviewed to determine if other sources would be pertinent and additional articles were abstracted if relevant.

The original search yielded 321 articles, from which 107 duplicates were excluded. A reviewer then screened titles and abstracts of the remaining 214 articles to determine suitability for inclusion. Articles that did not meet the criteria included those unrelated to the application of substances to the umbilical cord, articles focused on clinical trials comparing a variety of antiseptic applications to the umbilical cord, articles wherein the authors related only secondary data sources regarding umbilical cord-care practices, and articles that were unrelated to umbilical cord care, but had appeared in the search due to a common term, such as spinal cord. A total of 65 full-text articles were then reviewed using standardized inclusion criteria. The primary criterion for inclusion was a description of substances applied to the umbilical cord stump in the days following birth. Based on these criteria, a total of 46 of the 65 articles were included in this review. Secondary data about beliefs in relation to umbilical cord care and other cord-care practices were also recorded, if available. Data regarding cord-care practices were extracted from the articles using a standardized tracking form in Excel. Data items included:

- ○ What was used to cut the umbilical cord?
- ○ What was used to tie the umbilical cord?
- ○ Applications of a substance to the umbilical cord stump.
  - ○ What substance was applied?
  - ○ How often it was applied?
  - ○ How many days it was applied?
  - ○ Why was it applied (belief)?
  - ○ Who applied the substance?
  - ○ Source of product supply.
  - ○ Cost of product applied.
- ○ Other newborn skin-care practices, such as infant massage, that could contribute to the development of neonatal sepsis or tetanus were also tracked.

We synthesized data relating to cord-care practices and substance used on the cord by country. Because most of the studies were qualitative or observational in design, we were unable to draw any statistical comparisons. This review reporting followed the Preferred Reporting Items for Systematic Reviews and Meta-Analyses (known as PRISMA) reporting guidelines [6], as warranted.

## Results

**Fig. 1:**
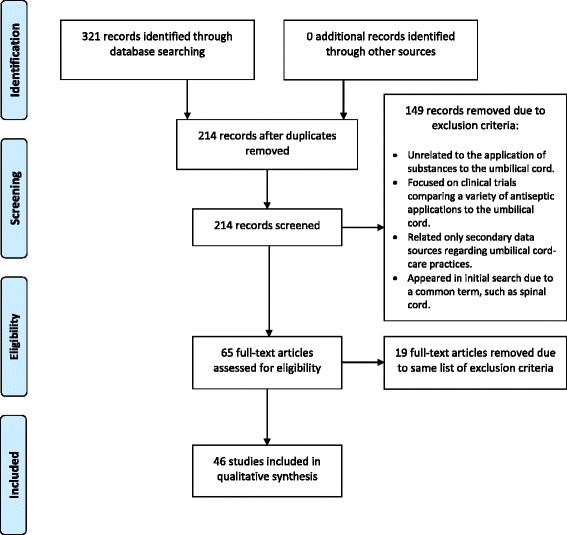
PRISMA flow diagram for this review article

A total of 46 articles were included in this review of umbilical cord-care practices. Figure 1 presents the flow diagram of the review process.

The 46 articles included data from 15 distinct low- and middle-income countries in sub-Saharan Africa (8 countries), Asia (5 countries), North Africa (1 country), and Latin America and the Caribbean (1 country). In sub-Saharan Africa, the majority of articles came from Uganda (6), followed by Tanzania (4), Ethiopia and Nigeria (3 each), Ghana and Zambia (2 each), and Benin and Sierra Leone (1 each). In Asia, the majority of articles came from Pakistan (7), followed by India and Nepal (5 each), Bangladesh (3), and Turkey (2). In North Africa, one article came from Egypt. In the Latin America/Caribbean region, one article came from Haiti. Table 1 provides general details about the articles included in this review. While country income classification was not a predetermined criteria for inclusion or exclusion, umbilical cord care articles from high-income countries were solely focused on the comparisons and uses of antiseptics and; therefore, none are included in this review based on the exclusion criteria.

**Table 1:** Articles included in this review, with study details

First author	Journal	Study type	Data collection year(s)	Country	Region	Rural/urban	Place of birth	Sample size
Abhulimhen-Iyoha [12]	Niger J Clin Pract. 2012	Cross-sectional survey	2009	Nigeria	Benin City	Urban	Community and facility	497 mothers.
Adejuyigbe [30]	Matern Child Nutr. 2008	Mixed-methods, cross-sectional	2003	Nigeria	Ekiti State	Rural	Community and facility	60 household surveys. 12 IDIs (healers, TBAs, birth home staff, nurses/midwives). 8 FGDs (total of 42 individuals. Elderly males and females). 4 questionnaires (fathers and grandmothers).
Alam [54]	J Perinatol. 2008	Cross-sectional, descriptive	2006	Bangladesh	Sylhet District	Not indicated	Community and facility	400 household surveys. 60 semi-structured interviews: 20 recently delivered women, 20 senior females, 10 husbands, 10 senior males.
Alhaji [37]	Niger Med J. 2013	Retrospective record review	2009–2010	Nigeria	North-eastern	Not indicated	Community and facility	51 cases of tetanus.
Alparslan [13]	Jpn J Nurs Sci. 2013	Descriptive and cross-sectional	2008	Turkey	Sivas	Urban	Not indicated	263 mothers.
Amare [14]	BMC Int Health Hum Rights. 2014	Qualitative	2013	Ethiopia	Oromia, Amhara, Tigray, and SNNPR (Sidama Zone)	Rural	Community and facility	IDIs with 32 total. One community in each region. Each study community: 6 mothers, 4 grandmothers, 2 TBAs, 2 HEWs.
Ayaz [55]	PLoS One. 2010	Community-based, cross-sectional	2006	Pakistan	Karachi (squatter settlement)	Urban	Community and facility	565 women between 28 to 33 days post-delivery.
Byaruhanga [7]	Midwifery. 2011	Qualitative	2008	Uganda	Kayunga, Soroti, and Ntugamo Districts	Rural	Community	6 IDIs with TBAs. 9 FGDs with 10 to 15 participants each (mothers, older females, and husbands).
Callaghan-Koru [56]	BMC Pediatr. 2013	Cross-sectional household survey of newborn care practices	2012	Ethiopia	Tigray, Oromiya, Amhara, and SNNPR	Not indicated	Community and facility	218 mothers.
Darmstadt [31]	Trop Med Int Health. 2007	Qualitative	2000	Egypt	Governorates of Aswan, Luxor, and Fayoum	Rural	Not indicated	217 households interviewed within 7 days of birth.
Degefie [16]	BMC Int Health Hum Rights. 2014	Qualitative	2012	Ethiopia	Sidama Zone of SNNP and East Shewa and West Arsi Zones of Oromia	Rural	Community and facility	8 KIIs with grandmothers. 27 IDIs with mothers. 7 IDIs with TBAs. 15 IDIs with fathers.
Dhingra [24]	BMC Pregnancy Childbirth. 2014	Qualitative	Not stated	Tanzania	Pemba Island	Urban and rural	Community and facility	11 FGDs and 80 IDIs with mothers, grandmothers, untrained and trained TBAs.
Falle [57]	J Health Popul Nutr. 2009	Qualitative	Unknown	Nepal	Sarlahi district, Southern Nepal	Rural	Community	93 TBAs.
Geçkil [25]	Midwifery. 2009	Descriptive	2004	Turkey	Adiyaman	Urban	Facility	273 mothers.
Ghosh [58]	J Biosoc Sci. 2010	Qualitative	2008	India	Uttar Pradesh	Peri-urban	Community and facility	892 women.
Gilani [59]	Rural Remote Health. 2014	Community-based, cross-sectional	2011	Pakistan	Muzaffarabad in Azad Jammu & Kashmir	Urban and rural	Community and facility	104 mothers of neonates with current illness or illness within previous 2 weeks.
Grant [60]	Paediatr Child Health. 2014	Qualitative, cross-sectional	2013	Uganda	Luwero district, Bamunanika sub-district	Rural	Not indicated	101 mothers or infant caregivers of deceased infants.
Gul [34]	Int J Health Sci (Qassim). 2014	Cross-sectional	2010	Pakistan	Karachi	Urban	Community and facility	170 mothers.
Herlihy [8]	PLoS One. 2013	Qualitative	2010	Zambia	Southern. Choma and Monze districts (rural) Livingstone and Mazabuka (urban)	Urban and rural	Community and facility	36 FGDs with grandmothers, breastfeeding mothers, midwives, TBAs. 42 IDIs with CHWs, community leaders, health professionals, religious leaders, TBAs, traditional healers.
Hill [15]	Pediatr Infect Dis J. 2010	Qualitative	2006–2007 (qualitative) 2008–2009 (quantitative)	Ghana	Brong Ahafo region	Rural	Community	9167 quantitative surveys of women who delivered between April 2008 and May 2009. 25 birth narratives. 30 IDS with women delivered in past 2 months. 2 FGDs with women delivered in past year or were pregnant. 20 IDIs and 6 FGDs with birth attendants/grandmothers. 12 IDIs and 2 FGDs with husbands.
Jennings [61]	Matern Child Health J. 2015	Pre-post randomized design; data drawn from interviews and observations	Unknown	Benin	Zou/Collines Region	Rural	Facility	411 mother-newborn pairs.
Karas [62]	J Trop Pediatr. 2012	Data collected within a community-based, cluster-randomized trial	2002–2006	Nepal	Sarlahi district, Southern Nepal	Rural	Community and facility	23662 infants.
Kayom [9]	Int J Pediatr. 2015	Cross-sectional descriptive	2012	Uganda	Kampala	Urban	Community and facility	335 mothers.
Kesterton [63]	BMC Pregnancy Childbirth. 2009	Mixed-methods	Quantitative (1996–1998), qualitative (2004–2005)	India	Karnataka	Rural	Community and facility	388 surveys of mothers. 39 IDIs with mothers, grandmothers, birth attendants, and key informants. 8 FGDs.
Khadduri [33]	J Perinatol. 2008	Qualitative	2000	Pakistan	Haripur District, NW Frontier Province	Rural and peri-urban	Community and facility	64 IDIs with mothers, fathers, TBAs. 34 FGDs.
Khan [10]	J Neonatal Perinatal Med. 2013	Cross-sectional	Unknown	Pakistan	Gilgit-Baltistan	Rural	Not indicated	708 mothers.
Kumar [64]	Lancet. 2008	Cluster-randomized, controlled efficacy trial	2006	India	Shivgarh, Uttar Pradesh	Rural	Community and facility	3400 mothers.
Madhu [32]	Indian J Community Med. 2009	Cross-sectional	2006	India	Kengeri, Karnataka	Rural	Community and facility	100 mothers.
Mangwi Ayiasi [65]	PLoS One. 2016	Randomized community intervention trial	2013–2014	Uganda	Masindi and Kiryandongo districts	Not indicated	Community and facility	1385 pregnant women.
Memon [66]	J Neonatal Perinatal Med. 2013	Cross-sectional KAP (knowledge, attitudes, and practices)	2005–2006	Pakistan	Matiari district, Sindh Province	Rural	Community and facility	1490 mothers.
Moran [16]	BMC Pregnancy Childbirth. 2009	Mixed-methods	2007	Bangladesh	Dhaka slums (Korali and Kamrangir Char)	Urban	Community and facility	36 women who were currently pregnant or those who had had at least one delivery.
Moyer [21]	BMC Pregnancy Childbirth. 2012	Qualitative	2010	Ghana	Northern. Kassena-Nankana districts	Rural	Community and facility	72 IDIs with women with newborns, health care providers, TBAs/herbalists, community leaders. Unspecified number of FGDs with grandmothers, compound heads, heads of household.
Mrisho [23]	Trans R Soc Trop Med Hyg. 2008	Qualitative	2005–2007	Tanzania	Lindi and Mtwara regions	Rural	Community and facility	40 IDIs with recently delivered women, pregnant women, TBAs. 16 FGDs with women who had given birth at least once.
Mullany [67]	Am J Epidemiol. 2007	Data collected within a community-based, cluster-randomized trial.	2002–2005	Nepal	Southern	Not indicated	Not indicated	15755 newborns.
Mullany [26]	Pediatr Infect Dis J. 2009	Data collected within a community-based, cluster-randomized trial.	2004–2005	Tanzania	Pemba Island	Rural	Community and facility	1653 infants (but only those born at home were evaluated for substances applied [1158]).
Raza [36]	J Postgrad Med. 2004	Case–control	1998–2001 (record review parameters)	Pakistan	Karachi (squatter settlement)	Urban	Not indicated	375 newborns.
Sacks [18]	BMC Pregnancy Childbirth. 2015	Qualitative	2010–2011	Zambia	Choma District	Rural	Community and facility	36 IDIs, 5 FGDs, and 8 observational sessions with recently-delivered women, TBAs, clinic and hospital staff.
Shamba [19]	J Health Popul Nutr. 2013	Qualitative	2008	Tanzania	Lindi Region	Rural	Community	11 birth narratives. 6 FGDs with women who delivered at home. 2 FGDs with birth attendants (any woman who assisted someone with a delivery in the last year).
Sharkey [11]	Health Policy Plan. 2016	Mixed-methods	2012	Sierra Leone	Kambia, Tonkolili, Kailahun, and Pujehun Districts	Rural	Community and facility	98 IDIs and 24 FGDs with pregnant women, caretakers, mothers, fathers, health care providers, CHWs, and TBAs. 6000 household surveys.
Sharma [28]	BMC Pregnancy Childbirth. 2016	Qualitative	2012	Nepal	Not indicated	Rural	Not indicated	5 IDIs and 14 FGDs with mothers, grandmothers, men, and health care workers.
Sreeramareddy [68]	BMC Pregnancy Childbirth. 2006	Cross-sectional survey	2006	Nepal	Western	Urban	Community	240 mothers.
Upadhyay [29]	Acta Paediatr. 2012	Cross-sectional survey	2010	India	Haryana (northern India)	Rural	Community and facility	415 mothers.
Waiswa [20]	BMC Pregnancy Childbirth. 2008	Qualitative	2006–2007	Uganda	Busoga (eastern Uganda)	Rural	Community and facility	10 FGDs with younger mothers (aged <30 years), older mothers with grandchildren, fathers, older children (up to age 13) who act as child minders. KIIs with 6 health workers and 4 TBAs.
Waiswa [69]	BMC Pregnancy Childbirth. 2010	Cross-sectional	2007	Uganda	Iganga-Mayuge Demographic Surveillance Site	Rural	Community and facility	393 mothers.
Walsh [22]	Glob Public Health. 2015	Qualitative	2013	Haiti	Petit-Gôave	Rural	Community and facility	5 FGDs with mothers with newborns, mothers with children under age two, and grandmothers and pregnant women, TBAs, CHWs, and traditional healers (voudou priests and priestesses).
Winch [27]	Lancet. 2005	Mixed methods	2001–2002	Bangladesh	Beanibazar, Zakiganj, and Kanaighat sub-districts in Sylhet District	Not indicated	Community and facility	39 IDIs with mothers, fathers, grandmothers, and TBAs. 6050 household surveys of mothers.

Abbreviations

*CHW* community health worker

*FGD* focus group discussion

*IDI* in-depth interview

*KII* key informant interview

*TBA* traditional birth attendant

### Beliefs

Beliefs related to the application of substances to the umbilical cord varies by country and by regions or cultural groups within a country. The intention behind applying a substance to the umbilical cord is to promote healing [7–11] and hasten the separation of the cord [7, 12–14] either by keeping the cord stump moist [10, 14, 15] or by drying it out [8, 16–20] to prevent pain/infection/bleeding [10, 12, 16, 17, 21], or to keep the “wind” (evil spirits) or cold/air [10, 16, 22] out of the infant. Table 2 provides an overview of cord-care practices described in each article included in this review.

**Table 2:** Articles included in this review, with cord care practices

First author	Journal	Country	Purpose of study and overall cord care practices (if stated)	What substance was applied to the cord
Abhulimhen-Iyoha [12]	Niger J Clin Pract. 2012	Nigeria	A study to determine the factors that influence cord care practices among mothers in Benin City, Nigeria.	Beneficial cord care (as defined by study authors): methylated spirits (20.5%), or non-beneficial substances: hot compress, herbs, native chalk, salt, sand, saliva, palm oil, menthol-containing balm, petroleum jelly, and toothpaste (substances were used alone or in combination and mainly applied at home) (76.9%).
Adejuyigbe [30]	Matern Child Nutr. 2008	Nigeria	Study examines differences in feeding and care between low-birthweight and normal-weight newborns. It is no clear from the article whether the cord care practices mentioned were specific to low-birthweight babies.	Fomentation with dry heat, application of white powder, and bandaging to prevent infection.
Alam [54]	J Perinatol. 2008	Bangladesh	An assessment of umbilical and skin care knowledge and practices for neonates in Sylhet District, Bangladesh. A boiled or new blade was used in over half the births (64%). Half of the families applied a substance to the cord.	Turmeric (83%), boric powder (53%), mustard oil, ash, Dettol, coconut oil, Nebanol ointment, ginger, chewed rice. Also practiced shek dewa (heat treatment of cord). Respondents did not indicate any concern about having clean hands or using a clean cloth during shek dewa.
Alhaji [37]	Niger Med J. 2013	Nigeria	Review of neonatal tetanus cases at Maiduguri Teaching Hospital.	Hot fomentation, charcoal, mixed, toothpaste, other.
Alparslan [13]	Jpn J Nurs Sci. 2013	Turkey	A study to identify traditional neonatal care practices applied by women ages 15 to 49. Cord cutting and tying practices were not discussed. Mothers reported putting something on the cord to make it separate faster, tying the belly with a rope, and also putting a buttered cloth over the infant’s infected belly.	This study reports on previous studies from Turkey which identified the following substances placed on the umbilical cord: dry coffee, olive oil, rotten tree powder, myrtle, sugared fat, hellebore, black sesame, and burnt cloth. This study did not inquire about specific substances, but did report that 10.3% of the women put a substance on the cord, 7.2% tied the belly with a rope, and 1.5% put a buttered cloth over the infant’s infected belly.
Amare [14]	BMC Int Health Hum Rights. 2014	Ethiopia	Investigation of practices and perspectives related to umbilical care in Ethiopia. A new razor blade which is sometimes boiled (22/24 mothers), cord tie varies (sewing thread, thread from kerosene stove, sisal, blanket strips, etc.). Cord is usually cut before delivery of placenta. Application of substance in most cases. Those who did not apply substance were either advised by health workers or said it was not customary (5/24 mothers).	Butter, petroleum jelly, hair lotion (Oromia). Butter or nothing (Amhara). Butter (Tigray). Petroleum jelly, hair lotion, or nothing (Sidama). Iodine or Gentian violet (applied by health extension workers in Oromia and Tigray). One health extension worker in Sidama advised application of tetracycline.
Ayaz [55]	PLoS One. 2010	Pakistan	A study to understand newborn care practices in a squatter settlement of Karachi, Pakistan. For home births, clean instruments were used to cut the cord in 53.2% of births, 0.4% reported unclean instruments (unclean blade, etc.), and 46.4% did not know. It is assumed that clean instruments were used for facility births. Substances were put on the cord in 57.7% of births regardless of place of birth.	Ointment (33%), ghee (27%), coconut oil (19%), mustard oil (9.5%). Some also applied surma (locally made kohl), clove oil, turmeric, and talcum powder.
Byaruhanga [7]	Midwifery. 2011	Uganda	A study to explore acceptability and feasibility of newborn care practices at the household and family level in rural Uganda. Dry cord care was stated as difficult to follow.	Herbs, onions, ash from burnt papyrus, petroleum jelly, powder, saliva, ghee, soot mixed with ghee, surgical spirit, water, butter.
Callaghan-Koru [56]	BMC Pediatr. 2013	Ethiopia	A study describing newborn care practices reported by recently-delivered women in four regions of Ethiopia. New string was used to tie cord in 46% of births, but fiber from the false banana (*ensete*) plant was also common. New razor or blade was used in home births to cut the cord 88% of the time and scissors were most commonly used in facilities. Women delivering at home applied a substance 27% of the time. While women who delivered in facilities stated that no substance was applied 47% of the time, 43% did not know if a substance had been applied.	Most commonly applied substance was butter. Other substances were applied, but not identified.
Darmstadt [31]	Trop Med Int Health. 2007	Egypt	Survey of home care practices during the first week of life in three rural Egyptian Governorates. Handwashing is not routine. Three-quarters of infants had a substance applied to their umbilical cords. Cord stump was also frequently covered by diaper.	Antiseptic (alcohol was used in 80% of antiseptic applications). Kohl (9%) was also used. Kohl contains lead.
Degefie [16]	BMC Int Health Hum Rights. 2014	Ethiopia	A study on the beliefs and practices related to immediate newborn and postnatal care in four rural communities in Ethiopia. Cord is typically cut with a new blade. Grandmothers and TBAs rub cord before cutting to prevent blood seeping out (not all wear gloves or wash hands before receiving baby). Cord usually tied with thread. Applications to the cord differ by region.	Ointment (Sidama) or butter (East Shewa).
Dhingra [24]	BMC Pregnancy Childbirth. 2014	Tanzania	An exploration of attitudes, beliefs, and practices related to delivery and newborn care in Pemba, Tanzania. The cord is cut soon after delivery. Clean cutting instrument appeared to be commonly understood among all participants (TBAs generally boil items in water). Cord tied with normal tailoring thread, but a few used special thread provided by the hospital. Cord is usually covered with a cloth to protect it from dust, flies, and mosquitos (and cord and surrounding area of male babies are also covered to prevent cord from falling on genitals). While the practice of dry cord care had reached all levels of society, substances were sometimes applied.	Saliva (*mate*), dirty door powder from old door (*ganda la popoo*), hot knife, charcoal powder, shells (*gamba la koa*), PPF powder, burning wood (*kijinga*), banana steam [sic] (*tojo*), fish bone. Substances were also applied AFTER cord fell off. Upon recognition of danger signs: hot knife, door dust, charcoal powder, sandalwood powder, ground sea shell, PPF or talcum powder, and fire steam.
Falle [57]	J Health Popul Nutr. 2009	Nepal	A study of the practices of traditional birth attendants working in Maithili-speaking (Madeshi) and Nepali-speaking (Pahadi) communities. About 27% of the TBAs reported applying a substance to the cord. New blade was used to cut cord in 89% of deliveries. Remaining 11% were cut with scissors (not clear if they were boiled or sterilized).	Mustard oil (64%), saliva (16%), antiseptics (12%).
Geçkil [25]	Midwifery. 2009	Turkey	A study of traditional cord care practices in southeastern Turkey. Practices included application of a substance, keeping the cord in a special place after it separated, and then discarding it in a place that would create significant meaning for the infant’s future.	Olive oil (37.7%), coffee, tar (15%).
Ghosh [58]	J Biosoc Sci. 2010	India	A study of antenatal, delivery, and postnatal practices of women in India who had experienced a neonatal death compared to current living children and also compared to women who had not experienced a neonatal death. Study showed better care practices for living children. Use of sterilized instrument for cutting cord was high across all groups (75% to 85%). Eight-six percent of the deceased infants had herbal paste or mustard oil applied to their umbilical cords (compared to 64% in the group of women with no neonatal deaths).	Herbal paste, mustard oil, other, antiseptic.
Gilani [59]	Rural Remote Health. 2014	Pakistan	A study to understand practices of mothers in regard to seeking healthcare for neonates in rural and urban settings of the capital district of Azad Jammu and Kashmir, Pakistan. The use of sterilized string to tie the cord was more common in urban areas vs. rural areas (44% vs. 27%). Use of a sterilized or boiled cutting tool was slightly more common in urban areas. Rural place of residence had a statistically significant association with use of unsterilized cutting and tying.	Not stated.
Grant [60]	Paediatr Child Health. 2014	Uganda	Article describes verbal autopsy procedure to determine probably causes of 72 neonatal deaths (30% occurred in first week of life) in Uganda. Sepsis due to infected umbilical cord was identified as the probable cause in 42% of deaths (highest of all identified causes).	Herbs, powder of papyrus reeds, soot from cooking pot, and saliva.
Gul [34]	Int J Health Sci (Qassim). 2014	Pakistan	A study to understand newborn care knowledge and practices among mothers in urban Pakistan. In 60% of home deliveries a new razor was used to cut the cord and 10% recalled the use of a household knife. Seventy-four percent (74%) or respondents applied a substance to the cord.	Coconut oil, mustard oil, ghee, olive oil, surma/kohl, turmeric, machine oil
Herlihy [8]	PLoS One. 2013	Zambia	A study to understand local perceptions of cord health and illness and the beliefs that shape cord care knowledge and practices. The cord was most frequently tied with white or black cotton knitting wool (home) or clamps (facility). In an emergency, women would use part of their *chitenge* (traditional fabric wrap). Traditional practice no longer used is the tying of the cord with *loozi* (fiber from the bark of a tree). A razor blade was the tool of choice for cutting the cord at home or scissors in a clinic. Despite the HIV epidemic, traditional healers and TBAs rarely sterilized tools, but CHWs and midwifes reported some type of disinfection (boiling or alcohol).	Different substances had different purposes. If cord is too brittle, cracking, bleeding, then a substance that increases softness (Vaseline®, cooking or motor oil, *mabono* (wild fruit) oil, or cream from sour milk). While warmed cooking or motor oil were rare, they were sometimes used as a base for other ingredients (charcoal, herbs). If cord takes too long to separate, items to dry it include: baby powder, charcoal dust, dried cow dung, dried chicken droppings, dust from threshold of home, ash from burnt pumpkin stem, crushed *loma* (wasps nest), or mud. Any of these would be pounded or ground into a fine powder. Medicinal substances: breast milk, python snake oil, banana, cow dung, *mukunku* (tree bark), traditional herbs, and dirt from pounding stick.
Hill [15]	Pediatr Infect Dis J. 2010	Ghana	A study exploring delivery practices in Ghana. The cord is generally cut with new razor blade (98%) and tied with new thread (90%), but dry cord care (the standard) was only practiced 8% of the time.	Hospital medicine (spirit) (31%), shea butter (47%), herbs (5%), other (16%). Also common to drip hot water on stump or apply a hot damp towel.
Jennings [61]	Matern Child Health J. 2015	Benin	A study focused on use of job aids to improve facility based postnatal counseling. The only discussion regarding cord care was in regard to substances applied.	Traditional substances include clay, ash, salt/spices, ground almonds.
Karas [62]	J Trop Pediatr. 2012	Nepal	An analysis of newborn care practices as part of a community-based trial investigating clean birthing kits. Although 97.7% of participants used the new razor blade with the kit, authors note that this is not customary practice. 95% waited until placenta was delivered to cut cord. Substance was applied to 18.8% of cords.	Mustard oil, antiseptic, ash, mud, other (other oil, breast milk, herbs/spices, saliva, and other substances).
Kayom [9]	Int J Pediatr. 2015	Uganda	A study to investigate newborn care practices in urban Uganda. Almost 70% of mothers had received teachings on cord care, but only 45% of those were from health professionals. Majority (89.6%) cleaned the cord twice a day. About 58% of the mothers in the study applied a substance to the cord.	Salty water, powder, Vaseline, spirit, normal saline, ripe banana (*gonja*), sap, soot, ash, saliva, herbs.
Kesterton [63]	BMC Pregnancy Childbirth. 2009	India	A study to understand local practices in rural Karnataka, India to improve implementation of essential newborn care practices. A sickle was used in about 1/3 of home births and a used blade in a majority of others. A substance is applied to the cord in some cases.	Turmeric, burning tip with castor oil lamp, antiseptic ointment.
Khadduri [33]	J Perinatol. 2008	Pakistan	A study to learn about maternal and newborn health knowledge and practices in rural Pakistan. A used blade, knife, or scissors was used often. The TBAs claimed to have washed the implements, but the mothers and father asserted that their TBAs had not washed the cutting instrument. Substances are applied to the cord using an uncleaned chicken feather either from the ground or freshly plucked.	Ghee and sometimes a paste of ghee with fried onions or mustard oil.
Khan [10]	J Neonatal Perinatal Med. 2013	Pakistan	A study to explore traditional newborn care beliefs and practices in Gilgit, Pakistan. The cord was cut with scissors (most common), blade (28%), or knife (5%). The cord was tied with available thread (55%) or a cord clamp (43.6%). The majority of households (77%) applied a substance to the cord.	*Matti* (crushed apricot seeds), cold cream, ghee, *haldi* (turmeric), mustard seed oil, Dettol, powder/wheat flour, antimony.
Kumar [64]	Lancet. 2008	India	Study conducted as part of a three-arm cluster randomized trial (control, newborn care package, and newborn care package + thermospot). Evaluated tying cord within 1/2 h of birth, cutting cord within 1/2 h of birth, cutting cord with clean blade, re-tying cord, application of substance on the cord and on the body.	Ash or clay on cord (60.9% of control group, 38% of ENC group, and 36.1% of ENC + TS group).
Madhu [32]	Indian J Community Med. 2009	India	A study to describe breastfeeding and other newborn care practices in rural Bangalore, India. In the small number of home deliveries (10% of total), a household knife was used to cut the cord in half (5 deliveries). Thirty-three percent of total births had something applied to the cord (regardless of place of birth).	Talcum powder or turmeric.
Mangwi Ayiasi [65]	PLoS One. 2016	Uganda	Study compared the effect of village health team (community health workers) visits and mobile phone consultations on maternal and newborn care practices. In the intervention group 60% of women used clean cord care practices while only 30% in the control group.	Baby powder was the most prevalent substance applied to the cord, followed by soot powder, herbal medicines, and animal dung.
Memon [66]	J Neonatal Perinatal Med. 2013	Pakistan	A study to assess knowledge, attitudes, and practices of women of reproductive age in rural Pakistan. Sixty-nine percent of participants applied a substance to the umbilical cord.	Mustard oil (54.4%), antimony (20.5%), and clarified butter (1.8%).
Moran [16]	BMC Pregnancy Childbirth. 2009	Bangladesh	A study to describe newborn care practices in the urban slums in Dhaka, Bangladesh. The cord is usually cut with a new or boiled blade. Thread used to tie the cord was generally not sterile. After cutting cord women reported cleaning the stump and surrounding areas (with wet cloth and soap or antiseptic liquid) and then applying heat and/or a substance to the cord.	Mustard oil, mustard with chopped, smashed garlic, coconut oil, boric powder, talcum powder, Savlon, *chular mati* (earth from a clay oven), homeopathic medicine. Also practiced *shek dewa* (heat treatment).
Moyer [21]	BMC Pregnancy Childbirth. 2012	Ghana	A study exploring clean birth practices and immediate newborn care in Ghana. The cord is typically cut with scissors or razor blade. Unprompted, women described the importance of sterilizing the tool. Cord was tied with rags, twigs, linen, string, rope, or plastic clamp. Substance were applied by 70% of women in the sample despite advice from providers to apply nothing.	Shea butter, ground shea nuts, local herbs, local oil, or “red earth sand”. Another person described application of juice from *pou* plant, spirits. Healthcare provider described practices no longer in use of using cow dung.
Mrisho [23]	Trans R Soc Trop Med Hyg. 2008	Tanzania	A study to understand childbirth and neonatal care practices. The cord usually cut with razor blade and tied with thread (or sometimes cut with millet stem). Razors generally not new. Substance applied to cord.	Traditional herbs + cooking oil or water that has been used to wash an adult woman’s genitals (*numbati*). Other substances include ash, breast milk, fluid from pumpkin flowers, and powder ground from local tree.
Mullany [67]	Am J Epidemiol. 2007	Nepal	An analysis of data on potential risk factors for omphalitis as part of a community-based, umbilical cord care trial. Substances were applied to the cord during the newborn period. Sometimes multiple substances were applied.	Mustard oil (80.4%), ash (7.1%), mud (6.8%), other substances (5.5%), non-study antiseptics (15.4%).
Mullany [26]	Pediatr Infect Dis J. 2009	Tanzania	Part of a larger clinical trial evaluating a suite of antenatal regimens and use of clean delivery kit in Tanzania. The cord was cut with items from a clean delivery kit. The study looked at two different definitions of cord infection (a broad definition and restrictive definition). Substances were applied to the cords in 10% of cases (home births only).	Dust, spirits/antibiotics, baby powder, charcoal, saliva, bark, vegetable oil, and dried roots; most common application was saliva.
Raza [36]	J Postgrad Med. 2004	Pakistan	Matched case control study of 125 cases of neonatal tetanus reported by 2 hospitals in Karachi between January 1998 and February 2001. There were 125 cases and 250 controls. Each case had two controls matched on gender, area of residency, time of birth within 2 months of case birth, and survived neonatal period and mothers had no history of tetanus toxoid immunization.	Mustard oil, ghee, surma (contains antimony and lead).
Sacks [18]	BMC Pregnancy Childbirth. 2015	Zambia	A study to understand local practices during the postnatal period. TBAs reported that they try to buy a new blade, but if there is no time to prepare then they use an old blade. Substances applied to the stump vary by whether child is classified (at birth) as “sick” or “healthy”. If there are blood clots in the umbilical cord, a sign of abnormality and future illness, the child is taken to a healer who treats the stump with herbs or the child is taken to a hospital. Until the cord separates, mother is expected to apply a wrap around the infant’s waist so that the cord does not touch the genitals (will make them infertile as an adult) and the cord is not supposed to fall on to the floor. Once the cord separates, the baby is bathed in cold water to make them strong.	Breast milk dripped onto cord. Full-term healthy babies: black powder (made from burn stem of pumpkin). Pre-term babies: green powder (ground dried roots of *mweeye* plant). This is considered gentler for preterm babies. If the appropriate herbs cannot be found then brick ash is used. Less common: after the cord falls off, fresh dried chicken dung is mashed and put onto the wound (rooster for male babies, hen for female babies). Participants explained that this tradition originated in Zimbabwe.
Shamba [19]	J Health Popul Nutr. 2013	Tanzania	A study to explore childbirth-related hygiene and newborn care practices in community deliveries in southern Tanzania. New blades were used in almost all instance, but new cord ties were rarely used. Substance was applied to cord by many mothers.	Breast milk, talcum powder, oil, petroleum jelly, ash, and dirt.
Sharkey [11]	Health Policy Plan. 2016	Sierra Leone	A study to understand maternal and neonatal care practices in four rural districts of Sierra Leone.	Pounded cassava.
Sharma [28]	BMC Pregnancy Childbirth. 2016	Nepal	A study to understand beliefs around childbirth and postnatal card in rural Nepal, particularly focusing on beliefs related to women being polluted following childbirth. A hasiya (scythe), a sickle, or a razor blade may be used to cut the umbilical cord and then a substance may be applied.	Antiseptic, cooking oil, ghee, plain water, toothpaste, and ash.
Sreeramareddy [68]	BMC Pregnancy Childbirth. 2006	Nepal	A study to understand reasons for delivering at home and about newborn care practices in urban areas of Nepal. The umbilical cord cut after delivery of placenta in 64.2% of cases, cut with new/boiled blade in 90.4% of cases (sickle, old knife, unboiled blade in 7.1%), no substance applied in 73.8% of cases. Newborn wrapped in old, washed cloth in 73.8% of cases.	Mustard oil (19.6%), oil + turmeric (2.1%), antiseptic (0.8%), unknown (9.6%), nothing applied (67.9%).
Upadhyay [29]	Acta Paediatr. 2012	India	A study to document neonatal care practices in the home and their associate with birth-attendant type. The cord was cut immediately after birth (50.6%) and cut after delivery after placenta in 29.6% (cutting immediately after birth was more common in skilled birth attendant deliveries). Clean instrument used in 70% and sterile tie used in 90%. Cord stump left dry in only 26.7%.	Antibiotic powder (46.4%), oil/ghee (42.8%).
Waiswa [20]	BMC Pregnancy Childbirth. 2008	Uganda	A study to explore the acceptability of evidence-based interventions to reduce maternal and newborn mortality in two rural districts in Uganda. Cord is usually cut with a new razor blade. Substances are applied.	Baby powder, spirit, herbs, soapy water, salty water.
Waiswa [69]	BMC Pregnancy Childbirth. 2010	Uganda	A study to explore the socioeconomic differences in the use of newborn care practices in Uganda. Most cords were cut with a clean razor blade and cords were tied with clean thread in the majority of cases. Half the mothers applied a substance to the cord.	Powder, surgical spirit, salty water, lizard droppings, ash, medicinal drugs.
Walsh [22]	Glob Public Health. 2015	Haiti	An exploration of current cord care practices in Petit-Goâve, Haiti. The cord needs to be covered by a cloth or substance to protect against evil spirits.	Burnt nutmeg, dirt from threshold of home, crushed charcoal, ash, burned cotton, palm oil, mixture of leaves and animal dung.
Winch [27]	Lancet. 2005	Bangladesh	A study to describe the beliefs surrounding the neonatal period in Sylhet District, Bangladesh. The cord is usually cut with a blade and then tied with a white thread, and then a substance is applied.	Chewed turmeric, chewed ginger, mustard oil with garlic, ash. *Shek dewa* (heat treatment) is also practiced.

*Abbreviation*

*TBA* traditional birth attendant

Table 3 illustrates the types of substances applied to the cord by country. The substance applied may depend upon the perceived nature of the cord. A moisturizing substance is applied if the cord is too brittle and a drying substance is applied if the cord takes too long to separate. For example, in southern Zambia, petroleum jelly or *mabono* (wild fruit) oil might be used if the cord is cracking or bleeding, while charcoal dust, baby powder, or burnt pumpkin stem may be used if the cord takes too long to separate [8]. In areas of Tanzania, Uganda, and Zambia [7, 8, 18, 20, 23], the infant cannot leave the home until the cord separates and/or the mother cannot return to her chores until that time. In the Tonkolili district of Sierra Leone, a traditional birth attendant noted the purpose of applying pounded cassava to the cord: “It will help the umbilical recover easily and the child will walk fast” [11].

**Table 3:** Substances used, by category

Category	Substances	Country/Countries
Oils	Clove oil [55]	Pakistan
	Coconut oil [17, 34, 54, 55]	Bangladesh, Pakistan
	Cooking oil (sometimes recycled) [8, 23, 28]	Nepal, Tanzania, Zambia
	*Mabono* (wild fruit) oil [8]	Zambia
	Machine/motor oil [8, 34]	Pakistan, Zambia
	Mustard oil [10, 17, 34, 36, 54, 55, 57, 58, 62, 66–68]	Bangladesh, India, Nepal, Pakistan
	Mustard oil with garlic [27]	Bangladesh
	Oil (unspecified) [19, 21, 29, 62]	Ghana, India, Nepal, Tanzania
	Oil with turmeric [68]	Nepal
	Olive oil [13, 25, 34]	Pakistan, Turkey
	Palm oil [12, 22]	Haiti, Nigeria
	Python oil [8]	Zambia
	Vegetable oil [26]	Tanzania
Herbs/spices/plants	Banana stem [24]	Tanzania
	Black sesame [13]	Turkey
	Burnt nutmeg [22]	Haiti
	Cassava [11]	Sierra Leone
	Dried roots [26]	Tanzania
	Ginger/chewed ginger [27, 54]	Bangladesh
	Hellebore [13]	Turkey
	Herbs (unspecified) [7–9, 12, 15, 20, 21, 23, 58, 60, 62]	Ghana, India, Nepal, Nigeria, Tanzania, Uganda, Zambia
	*Kijinga* (burning wood) [24]	Tanzania
	*Mukunku* (bark of a tree) [8]	Zambia
	Mustard with chopped, smashed garlic [17]	Bangladesh
	Myrtle [13]	Turkey
	*Pou* plant (juice) [21]	Ghana
	Pumpkin flowers (fluid) [23]	Tanzania
	Sap [9]	Uganda
	Spices (unspecified) [61, 62]	Benin, Nepal
	Turmeric/chewed turmeric [10, 27, 32, 34, 54, 55, 63]	Bangladesh, India, Pakistan
Minerals/powders	Antimony [10, 66]	Pakistan
	Ash [9, 19, 22, 23, 27, 28, 54, 61, 62, 64, 67, 69]	Bangladesh, Benin, Haiti, India, Nepal, Tanzania, Uganda
	Ash from burnt papyrus [7]	Uganda
	Bark [26]	Tanzania
	Black powder (burnt pumpkin stem) [8, 18]	Zambia
	Boric powder [17, 54]	Bangladesh
	Brick ash [18]	Zambia
	Chalk [12]	Nigeria
	Charcoal [8, 22, 24, 26, 37]	Haiti, Nigeria, Tanzania, Zambia
	*Chular mati* (earth from clay oven) [17]	Bangladesh
	Clay [61, 64]	Benin, India
	Dirt from pounding stick [8]	Zambia
	Dust/dust from threshold of home [8, 22, 24, 26]	Haiti, Tanzania, Zambia
	*Ganda la popoo* (dirty door powder from old door) [24]	Tanzania
	Green powder (ground dried roots of *mweeye* plant) [18]	Zambia
	Ground sea shell [24]	Tanzania
	Mud [8, 62, 67]	Nepal, Zambia
	Powder (unspecified) [7, 9, 10, 69]	Pakistan, Uganda
	Powder from ground local tree [23]	Tanzania
	Powder of papyrus reeds [60]	Uganda
	PPF powder (pyriproxyfen pesticide) [24]	Tanzania
	Red earth sand [21]	Ghana
	Rotten tree powder [13]	Turkey
	Salt [12, 61]	Benin, Nigeria
	Sandalwood powder [24]	Tanzania
	Soil/dirt/sand [12, 19]	Nigeria, Tanzania
	Soot/soot from cooking pot [9, 60, 65]	Uganda
	White powder [30]	Nigeria
Dung	Animal (unspecified) [65]	Uganda
	Animal (unspecified) mixed with leaves [22]	Haiti
	Chicken [8, 18]	Zambia
	Cow [8]	Zambia
	Lizard [69]	Uganda
Water	Hot water [15]	Ghana
	*Numbati* (water that has been used to wash an adult woman’s genitals) [23]	Tanzania
	Plain water [7, 28]	Nepal, Uganda
	Salty water [9, 20, 69]	Uganda
	Soapy water [20]	Uganda
Bodily fluids	Breast milk [8, 18, 19, 23, 62]	Nepal, Tanzania, Zambia
	Saliva [7, 9, 12, 24, 26, 57, 60, 62]	Nepal, Nigeria, Tanzania, Uganda
Food	Banana [8, 9]	Uganda, Zambia
	Butter [7, 14, 16, 56]	Ethiopia, Uganda
	Chewed rice [54]	Bangladesh
	Cream from sour milk [8]	Zambia
	Dry coffee [13, 25]	Turkey
	Fat and sugared fat [13]	Turkey
	Ghee [7, 10, 28, 29, 33, 34, 36, 55, 66]	India, Nepal, Pakistan, Uganda
	Ghee mixed with fried onions or mustard oil [33]	Pakistan
	Ghee mixed with soot [7]	Uganda
	Ground almonds [61]	Benin
	Ground shea nuts [21]	Ghana
	*Matti* (crushed apricot seeds) [10]	Pakistan
	Onion [7]	Uganda
	Wheat flour [10]	Pakistan
Personal care/medical	Alcohol [31]	Egypt
	Antibiotic powder [29]	India
	Antiseptic ointment [63]	India
	Antiseptics (unspecified) [26, 28, 31, 57, 58, 62, 67, 68]	Egypt, India, Nepal, Tanzania
	Baby powder/talcum powder [8, 17, 19, 20, 24, 26, 32, 55, 65]	Bangladesh, India, Pakistan, Tanzania, Uganda, Zambia
	Cold cream [10	Pakistan
	Dettol [10, 52]	Bangladesh, Pakistan
	Gentian violet [14]	Ethiopia
	Hair lotion [14]	Ethiopia
	Herbal medicine (unspecified) [65]	Uganda
	Homeopathic medicine (unspecified) [17]	Bangladesh
	Iodine [14]	Ethiopia
	Kohl/surma (can contain lead and antimony) [31, 34, 36, 55]	Egypt, Pakistan
	Medicinal drugs (unspecified) [69]	Uganda
	Menthol-containing balm [12]	Nigeria
	Methylated spirit [12]	Nigeria
	Nebanol ointment [52]	Bangladesh
	Ointment (unspecified) [16, 55]	Ethiopia, Pakistan
	Petroleum jelly [7–9, 12, 14, 19]	Ethiopia, Nigeria, Tanzania, Uganda, Zambia
	Savlon [17]	Bangladesh
	Shea butter [15, 21]	Ghana
	Surgical spirit [7, 9, 15, 20, 26, 69]	Ghana, Tanzania, Uganda
	Toothpaste [12, 28, 37]	Nigeria, Nepal
Other	Burnt cloth/burned cotton [13, 22]	Haiti, Turkey
	Fish bone [24]	Tanzania
	*Gamba la koa* (shells) [24]	Tanzania
	*Loma* (crushed wasps nest) [8]	Zambia
	Tar [25]	Turkey
	“Other substances” [15, 56, 58, 62, 67]	Ethiopia, Ghana, India, Nepal
Heat	Burning tip of cord [63]	India
	Fire steam [24]	Tanzania
	Hot fomentation or *shek dewa* (dry or moist heat) [12, 15, 17, 27, 30, 37, 54]	Bangladesh, Ghana, Nigeria
	Hot knife [24]	Tanzania

The substances applied may also vary by the infant’s perceived gestational age or state of health. For example, in the Choma District in Zambia, the substance applied to a newborn’s umbilical cord differs by the newborn’s perceived gestational age, as substances used for full-term babies are considered too strong for preterm babies. A black powder made from the burnt stem of the pumpkin plant is applied to the umbilical cord of full-term infants while a green powder made from the dried roots of the *mweeye* plant is applied to the cord of preterm/small infants as it is considered to be gentler than the black powder [18]. Also in southern Zambia, substances such as petroleum jelly, *mabono* (wild fruit) oil, cooking/motor oil, charcoal, dried cow dung or chicken droppings, burnt pumpkin stem, and crushed *loma* (wasps’ nest) are applied to the cord of the healthy infant. However, a separate set of substances that are considered to be medicinal are applied if the cord is red or if pus appears. The substances that are considered to be medicinal include: python oil, breast milk, alcohol, banana, cow dung, *mukunku* (bark of a tree), traditional herbs, or dirt from a pounding stick [8].

The length of the cord may have specific importance. In southern Zambia, it is believed that if the cord is too long, it will take too long to heal, and if it is too short, “the air will go in and this will make the baby die” [8]. The length of the cord is also believed to indicate the length of the genitals in both men and women [8]. Further, in southern Zambia and on the island of Pemba in the Zanzibar archipelago, Tanzania, the umbilical cord may be wrapped or bound with a strip of cloth to prevent it from touching the groin area [8, 18, 24], which is sometimes believed to cause infertility as an adult [18]. In areas of Zambia and Tanzania, where it is important to protect the cord from being taken by someone who wishes the infant or the family ill, the cord, and often the placenta, are disposed of through burial in a sacred place or by burning, or are sometimes put in a pit latrine/toilet to prevent them from being unearthed [8, 23]. In southeastern Turkey, the cord may be buried in a special place to help define the child’s path in life, such as burying the cord at a mosque or a school to help the child grow up to be a religious or educated person, respectively [25].

### Frequency of application

Few articles reported on the frequency of application of the substance (either the number of days or the number of times per day the substance was applied). In Ethiopia, the substance is applied one to three times per day up to the seventh day of life. However, the applications may not begin until the newborn is two or three days old [14]. In the Brong Ahafo region of Ghana, a substance is applied anywhere from every 30 min to 3 times per day [15]. In urban Uganda, mothers reported cleaning the cord with a substance at least twice per day [9]. On Pemba Island in the Zanzibar archipelago, Tanzania, multiple substances were applied to the umbilical area beginning on the sixth day after birth. In this study of more than 1000 infants born at home, only 10% (*n* = 109) had a substance applied to the umbilical cord and less than 11% of those applications were made in the first 48 h of life [26]. A study in Sylhet District in Bangladesh, involving 39 in-depth interviews of mothers, fathers, grandmothers, and traditional birth attendants and data from more than 6000 household surveys of mothers, found that substances were applied until the cord separated and that either turmeric or ginger are applied at birth and then a combination of mustard oil and garlic are applied twice daily until the separation of the cord [27].

Few studies identified who usually applied the substance to the cord. In the 19 studies where it was reported, either the mother or grandmother of the infant [9, 12–19, 21, 25, 27–32] or a senior woman [15, 19, 33] in the household applied the substance to the cord. In only a very few cases did a traditional birth attendant or health worker [11, 14, 19] apply a substance to the cord.

### Cost and source of substance

Few articles investigated the source of the substances applied to the umbilical cord and none reported on the cost of the substances applied. Shea butter or cooking oil were purchased from the market [15, 18]. One study reported that the cooking oil applied to the cord is generally a recycled product bought at the local market that had been previously used [18].

## Discussion

This study presents a view of traditional cord-care practices as reported during the last fifteen years in low- and middle-income countries. The desire to care for the umbilical cord of an infant appears to be universal in all cultures. Of interest is the description of the range of products applied to the newly cut cord and that substances are applied to the umbilical cord to infants born at home as well as in facilities. Participants in studies from Petit-Gôave, Haiti, and Karachi, Pakistan, reported that if an infant was born in a health facility, a substance would be applied to the umbilical cord upon returning home [22, 34].

The possible harm of these substances has not been fully quantified. In some cases, such as with kohl and surma used in Egypt and Pakistan, the lead and antimony included in the product is most likely harmful. Anecdotal literature often refers to the use of dung as a harmful traditional practice. In contrast, we found use of chicken/lizard/cow dung reported in only three countries (Haiti, Uganda, and Zambia), thereby suggesting that this practice is not as widespread as depicted or that it, as reported by a traditional birth attendant in northern Ghana, was practiced in the past and has since ceased [21]. It is also possible that application of preparations using dung is underreported in published literature. For example, unpublished formative research from the Kenieba and Koutiala Districts of Mali report use of *bassa bo* (lizard excrement powder mixed with shea butter) and *bagani dji* (insect powder mixed with shea butter, sap, or powder of *pourghère*) to help the stump fall off and heal the umbilicus [unpublished data from formative research conducted in Mali in 2015 by the Maternal Child Survival Program of the US Agency for International Development]. Data from the Kita and Diema Districts in Mali report application of cow dung, shea butter, ash, alcohol, and Maggi cubes to the umbilical cord [unpublished data from endline survey conducted in Mali in 2014 by the Maternal and Child Health Integrated Program, predecessor to Maternal Child Survival Program].

Other product categories (i.e., oils, herbs/spices/plants, mineral/powder, water, bodily fluids, food, personal care/medical products) and processes such as heat treatment may or may not be harmful and could warrant further investigation. Neonates and young infants are more prone to infection than older children and adults and evidence suggests that their immune systems are still developing rather than being fully formed at the time of birth. Further, newborns appear to be particularly vulnerable to the types of intracellular pathogens that commonly cause neonatal sepsis [35], such as some strains of streptococcus, *Escherichia coli*, and *Listeria monocytogenes*.

Concern has been expressed about transmission of HIV from mother to child through the application of breast milk to the umbilical cord [18]. Also, the use of *numbati* (water that has been used to wash an adult woman’s genitals) in Tanzania [23], could potentially pose a risk for transmission of HIV or other diseases. The application of unhygienic substances to or around the umbilical cord stump has been linked to tetanus in infants [2, 13, 25, 36–39]. *Clostridium tetani* bacteria, found in soil, dust, saliva, animal dung, and other sources, are the cause of tetanus infection [40].

Some substances, which are known to be harmful in other contexts, pose an unclear risk when applied to the umbilical cord. For example, motor/machine oil contains high levels of chemical additives and, once used for its intended purpose, can contain high levels of heavy metals and other minerals [41, 42]. While used motor/machine oil is well known to be harmful to the environment and causes dermatitis through long-term exposure, the health risks due to short-term exposure through an open wound is not well defined. Boric acid powder is used as a pesticide and has caused seizures and death in infants when ingested [43], but also has known antifungal properties and has been used as a treatment for conditions such yeast infections [44]. However, sources differ on whether it is safe to use boric acid on open wounds. Pyriproxyfen powder, a pesticide, poses minimal risk to humans in small quantities [45]. Other substances, such as oils, herbs, plants, food products, and heat treatments applied to the cord may be harmful depending on whether they have been contaminated in some way, such as through unhygienic preparation or storage.

This critical information regarding traditional cord-care practices can serve as the stepping stone to behavior change. We focused our review on a comprehensive description of reported cord-care practices with the intention of employing the knowledge to develop behavior-change strategies to support introduction of novel cord-care regimens, particularly 7.1% chlorhexidine digluconate for umbilical cord care. Randomized controlled trials investigating the use of 7.1% chlorhexidine digluconate for umbilical cord care have been conducted in Nepal [46], Bangladesh [47], and Pakistan [48]. A meta-analysis of the three studies demonstrated that application of chlorhexidine to the umbilical cord of the newborn led to a 23% reduction in all-cause neonatal mortality and a reduction in omphalitis ranging from 27 to 56% compared to control group depending on severity of infection [49]. Further, the World Health Organization recommends the application of 7.1% chlorhexidine digluconate (gel or solution) to the umbilical cord of neonates who are born at home in settings with high neonatal mortality or to replace the use of a harmful, traditional substances [50]. Despite previously reported substantial reductions in South Asia, results from recent trials in Zambia [51] and Tanzania [52] show that application of 7.1% chlorhexidine to the umbilical cord did not significantly reduce neonatal mortality rates in the study sites. This suggests that programmatic context and level of risk in the population as well as cord care practices must be considered in any behavior change initiative.

Findings from this review suggest that documentation of cord-care practices is not consistent throughout low- and middle-income countries. Given the heterogeneity of practices described in the literature, it is not clear if data from one country in a region also pertains to other surrounding countries and/or nearby ethnic groups. Overall, however, existing literature depicts a firm tradition of umbilical cord care in every culture. The desire to take some kind of action to address the newly cut umbilical cord seems to be a strong human desire. Participants in several studies noted that adhering to dry cord care was very difficult. For example, in Ghana, there was a belief that applying nothing to the cord would delay separation, cause discomfort, and potentially cause death for the infant by preventing the sore from healing and causing a sickness in the stomach [15]. In Uganda, the practice of dry cord care was noted as difficult to follow as it delays the cord separation and the return of the mother to chores [7]. This desire to actively care for a newborn could be utilized as a trigger for behavior change. For example, as applied in the Health Belief Model [53], knowledge of effective cord care (i.e., product, application procedure, number of days to use, number of times/day to apply) could function as a “cue to action” to allow caretakers to act in a positive manner.

This review has limitations, as noted. Reporting is not global in nature as the review was limited to sources published in English in peer-reviewed journals. Few of the studies conducted have been in-depth, qualitative assessments of umbilical cord care practices; therefore, much of the detailed information about beliefs and practices surrounding umbilical cord care comes from a few sources or countries, such as Tanzania and Zambia. This could lead to the assumption that some countries place greater importance on umbilical cord care practices than other countries where the data collection focused on a variety of newborn care practices. The paucity of data from Latin America and the Caribbean reflected in this review could stem from our decision to include only English-language material. Also, it is likely that additional anecdotal information is available in gray literature.

Additional research around cost and source of products used in cord care practices could assist programs that are targeting newborn care behavior change. Likewise, deeper ethnographic and/or qualitative investigation into the underlying meaning and significance of traditional cord-care practices could assist in formulating key messages being used to generate demand for novel prophylactic products.

## Conclusions

Findings from this review suggest that documentation of cord-care practices is not consistent throughout low- and middle-income countries, yet existing literature depicts a firm tradition of umbilical cord care in every culture studied. Cord-care practices vary by country and by regions or cultural groups within a country and employ a wide range of substances. The desire to promote healing and hasten cord separation are the underlying beliefs related to application of substances to the umbilical cord. The frequency of application of the substance (either the number of days or the number of times per day the substance was applied) and source and cost of products used are not well characterized. This desire to actively care for the umbilical cord of a newborn could be utilized to promote positive behavior change such as the introduction of 7.1% chlorhexidine digluconate for umbilical cord care. The variety in cord care practices and beliefs noted in this review however points toward the need to contextualize any behavior change approach to align with the local culture.

## Additional file

Additional file 1: Search Terminology. Exact narrative sequences used for literature search. (DOCX 12 kb)
