# Supplementary material for: Umbilical cord-care practices in low- and middle-income countries: a systematic review
Source: BMC Pregnancy Childbirth. 2017 Feb 20;17:68. doi: 10.1186/s12884-017-1250-7 (PMC5319165; doi:10.1186/s12884-017-1250-7)
Supplement: Additional file 1: — Search Terminology. Exact narrative sequences used for literature search. (DOCX 12 kb) [file 12884_2017_1250_MOESM1_ESM.docx]

**Additional File 1: Search terminology**

(("umbilical cord"[MeSH Terms] OR ("umbilical"[All Fields] AND "cord"[All Fields]) OR "umbilical cord"[All Fields]) AND care[All Fields] AND practices[All Fields]) AND (("2000/01/01"[PDAT] : "2016/08/24"[PDAT]) AND English[lang])

umbilical cord care[Title/Abstract] AND (("2000/01/01"[PDAT] : "2016/08/24"[PDAT]) AND English[lang])

(("umbilicus"[MeSH Terms] OR "umbilicus"[All Fields] OR "umbilical"[All Fields]) AND cord application[Title/Abstract]) AND (("2000/01/01"[PDAT] : "2016/08/24"

umbilical cord care practices[Title/Abstract] AND (("2000/01/01"[PDAT] : "2016/08/24"[PDAT]) AND English[lang])

(((((((cord[All Fields] AND care[All Fields] AND practices[All Fields]) NOT ("hematopoietic system"[MeSH Terms] OR ("hematopoietic"[All Fields] AND "system"[All Fields]) OR "hematopoietic system"[All Fields] OR "hematopoietic"[All Fields])) NOT ("brain diseases"[MeSH Terms] OR ("brain"[All Fields] AND "diseases"[All Fields]) OR "brain diseases"[All Fields] OR "encephalopathy"[All Fields])) NOT ("thyroid gland"[MeSH Terms] OR ("thyroid"[All Fields] AND "gland"[All Fields]) OR "thyroid gland"[All Fields] OR "thyroid"[All Fields] OR "thyroid (usp)"[MeSH Terms] OR ("thyroid"[All Fields] AND "(usp)"[All Fields]) OR "thyroid (usp)"[All Fields])) NOT ("rehabilitation"[Subheading] OR "rehabilitation"[All Fields] OR "rehabilitation"[MeSH Terms])) NOT ("spine"[MeSH Terms] OR "spine"[All Fields])) NOT spinal[All Fields]) NOT ("blood transfusion"[MeSH Terms] OR ("blood"[All Fields] AND "transfusion"[All Fields]) OR "blood transfusion"[All Fields] OR "transfusion"[All Fields]) AND (("2000/01/01"[PDAT] : "2016/08/24"[PDAT]) AND English[lang])
